# Type 2 Diabetes Mellitus and Osteoporosis: Site-Specific Bone Mineral Density Variations and Metabolic Correlations in Postmenopausal Saudi Women

**DOI:** 10.3390/medicina61050789

**Published:** 2025-04-24

**Authors:** Nogood Mashahi Alhowiti, Amal M. H. Mackawy, Wanian Mohammed Al Wanian, Mohammad Alshebremi, Khaled S. Allemailem, Hajed Obaid Abdullah Alharbi

**Affiliations:** 1 Department of Medical Laboratories, College of Applied Medical Sciences, Qassim University, Burydah 51452, Saudi Arabia; 381225472@qu.edu.sa (N.M.A.); w.alwanian@qu.edu.sa (W.M.A.W.); m.alshebremi@qu.edu.sa (M.A.); k.allemailem@qu.edu.sa (K.S.A.); 2 Department of Medical Biochemistry and Molecular Biology, Faculty of Medicine, Zagazig University, Zagazig City 7120730, Egypt

**Keywords:** type 2 diabetes mellitus, osteoporosis risk, bone mineral density, health of postmenopausal women, estrogen, parathyroid hormone, cross-sectional study, osteoporosis association factors

## Abstract

*Background and Objectives:* Osteoporosis (OP) is a prevalent condition among postmenopausal women, with an estimated 40% of Saudi women affected. Concurrently, type 2 diabetes mellitus (T2DM) is highly prevalent in the Qassim region, affecting 45% of individuals aged 40 and older. Despite conflicting evidence regarding the impact of T2DM on bone health, its role in OP development remains uncertain. *Materials and Methods:* This study investigates site-specific bone mineral density (BMD) variations and their metabolic correlations in postmenopausal Saudi women with T2DM. A cross-sectional study included 250 postmenopausal Saudi women, 100 without diabetes (Group 1) and 150 with diabetes (Group 2), matched for age, menopausal duration, and body mass index (BMI). BMD at the femoral neck (FN) and lumbar spine (LS) was assessed using dual-energy X-ray absorptiometry (DXA). Biochemical markers, including parathyroid hormone (PTH), alkaline phosphatase (ALP), estrogen, calcium, and HbA1c, were assessed. Statistical analyses, including chi-square tests, *t*-tests, ANOVA, Pearson correlation, and multivariate regression, evaluated BMD variations and biochemical associations. *Results:* Patients with diabetes exhibited significantly higher FN T-scores than those without diabetes (*p* = 0.001), while LS T-scores showed no significant difference. BMD distribution (normal, osteopenia, OP) did not differ between the groups (*p* > 0.05). FN T-scores correlated positively with parathyroid hormone (PTH) and alkaline phosphatase (ALP) levels, reduced estrogen, and prolonged menopause duration (*p* < 0.01) but were inversely associated with estrogen levels and menopause duration (*p* < 0.01). *Conclusions:* No significant association was found between HbA1c and BMD. Additionally, BMI demonstrated a protective effect on FN BMD. T2DM appears to influence bone metabolism without directly causing OP in postmenopausal women. Aging, menopause duration, metabolic markers (PTH, ALP, estrogen), and BMI play crucial roles in BMD variations, with a protective effect of BMI. These findings underscore the importance of site-specific BMD assessment and metabolic profiling in postmenopausal women with diabetes. Further longitudinal research is needed to elucidate the underlying mechanisms affecting bone health in postmenopausal women with diabetes.

## 1. Introduction

Osteoporosis (OP) and type 2 diabetes mellitus (T2DM) are two of the most prevalent chronic conditions affecting postmenopausal women worldwide, and both of them pose significant risks for fractures and metabolic complications [1,2]. OP is characterized by reduced bone mineral density (BMD) and deterioration of bone microarchitecture, leading to an increased risk of fractures [3]. T2DM, a metabolic disorder primarily driven by insulin resistance, has been associated with alterations in bone metabolism, with growing evidence suggesting that patients with diabetes have an increased risk of fragility fractures despite normal or higher BMD values [4,5].

In Saudi Arabia, the prevalence of osteoporosis is alarmingly high, affecting approximately 40% of postmenopausal women, with regional variations influenced by genetic, dietary, and lifestyle factors [6]. Simultaneously, the burden of T2DM continues to rise, with estimates indicating that nearly 45% of individuals aged 40 and older in the Qassim region have diabetes [7]. The complex interplay between these two conditions remains a subject of ongoing debate, with conflicting reports regarding the impact of T2DM on bone health. While some studies suggest that hyperinsulinemia may exert anabolic effects on bone mass, others highlight the detrimental impact of chronic hyperglycemia and advanced glycation end-products (AGEs) on bone quality and strength [8,9].

In postmenopausal women, estrogen deficiency plays a critical role in bone resorption, exacerbating the risk of osteoporosis, particularly among individuals with diabetes, who may experience further dysregulation of bone remodeling markers such as parathyroid hormone (PTH) and alkaline phosphatase (ALP) [10]. Despite these known mechanisms, the precise relationship between T2DM, hormonal alterations, and BMD loss remains insufficiently understood, particularly in Middle Eastern populations, where genetic and environmental factors may contribute uniquely to disease progression [11,12].

This study aims to evaluate the association between T2DM and osteoporosis among postmenopausal Saudi women in the Qassim region, with a particular focus on BMD variations at the femoral neck (FN) and lumbar spine (LS). Additionally, we explore the role of key metabolic and hormonal markers, including PTH, ALP, and estrogen, to elucidate their contributions to bone health in postmenopausal women with and without diabetes. Understanding these associations will help develop more targeted prevention and intervention strategies for osteoporosis in high-risk populations.

## 2. Methodology

### 2.1. Study Design and Population

This cross-sectional study assessed the association between T2DM and OP in postmenopausal Saudi women residing in the Qassim region, Kingdom of Saudi Arabia. The study population consisted of 250 postmenopausal women, including 150 women diagnosed with T2DM (diabetes group; group I) with a mean age ± S.D of 58.47 ± 6.07 and 100 controls without diabetes with a mean age ± S.D of 57.13 ± 6.77, named group II. Female patients with diabetes were recruited from King Fahd Specialist Hospital (KFSH), Diabetic and Endocrinology Centre, and Buraidah Central Hospital in Buraidah City, Qassim region, Kingdom of Saudi Arabia (KSA). Before enrollment, participants provided informed consent after being thoroughly briefed on this study’s primary objectives and the procedures for sample collection. Participants were selected through a randomization process, ensuring that the inclusion criteria were met.

To control for potential confounding variables, the participants were matched based on age, duration of menopause, and BMI.

- Inclusion Criteria: This study included postmenopausal Saudi women aged ≥ 45 years with at least 12 months of amenorrhea, indicating the onset of menopause. Women diagnosed with type 2 diabetes mellitus (T2DM) were selected based on the American Diabetes Association (ADA) criteria: HbA1c ≥ 6.5% or fasting plasma glucose ≥ 126 mg/dL. Additionally, women without diabetes with normal fasting glucose levels (i.e., <100 mg/dL) were included. All participants had to provide informed consent and express willingness to partake in this study.
- Exclusion Criteria: Women with type 1 diabetes mellitus, gestational diabetes, a history of malignancies, thyroid disorders, chronic kidney disease, or primary/secondary hyperparathyroidism were excluded. Also excluded were women on medications that could influence bone metabolism, such as corticosteroids, bisphosphonates, hormone replacement therapy, or thiazolidinediones, as well as those with metabolic bone diseases or autoimmune disorders affecting bone health. By rigorously selecting participants, this study aimed to minimize confounding factors and ensure that the results accurately reflect the effects of T2DM and menopause on bone health in postmenopausal women.

### 2.2. Informed Consent

This study adhered to the ethical principles outlined in the Declaration of Helsinki and obtained informed consent from all participants before their involvement. Each participant was provided with comprehensive information regarding this study’s objectives, significance, and procedures before completing the questionnaire and participating in the research. Informed consent was obtained through an online consent forms, ensuring that participants understood the full scope of this study. All personal information collected was kept strictly confidential and used solely for this study. Additionally, participants were fully informed of their right to withdraw from this study at any time without any consequences.

### 2.3. Ethical Considerations

The study protocol adhered to the principles outlined in the Declaration of Helsinki and was approved by the Institutional Review Board (IRB) of Qassim University and the Regional Research Ethics Committee (NO -1442-2136242). Written informed consent was obtained from all participants before data collection.

### 2.4. Data Collection and Measurements

- Anthropometric and Clinical Assessments

All participants underwent standardized anthropometric and clinical assessments conducted by trained healthcare professionals. Data on age, menopausal duration, and medical history were collected through structured interviews. BMI was calculated as weight (kg) divided by height squared (m^2^), while waist and hip circumferences were measured using a flexible measuring tape. Blood pressure (BP) was measured using an automated sphygmomanometer in a seated position after five min of rest.

- Bone Mineral Density (BMD) Assessment

BMD was evaluated using dual-energy X-ray absorptiometry (DXA) at two anatomical sites: the lumbar spine (LS) and femoral neck (FN). DXA scans were performed using a GE Lunar Prodigy DXA system following standard manufacturer protocols. The DXA was performed on a Densitometer Prodigy Series X-Ray Tube Housing Assembly, Mark 8743 BX-1L, produced by General Electric Company (GE) in 2010, Madison, WI, USA. BMD results were expressed as T-scores and classified according to World Health Organization (WHO) criteria as normal (T-score ≥ −1.0), osteopenia (−2.5 < T-score < −1.0), or osteoporosis (T-score ≤ −2.5) [12].

- Diagnosis of Type 2 Diabetes Mellitus (T2DM)

T2DM was diagnosed based on the American Diabetes Association (ADA) 2007 criteria: fasting plasma glucose (FPG) ≥ 126 mg/dL, 2 h plasma glucose (PG) ≥ 200 mg/dL, or HbA1c ≥ 6.5%.

- Blood Sample Collection and Storage

Blood samples were collected after an overnight fast of 8 h. A total of 10 mL of blood was drawn to measure bone biochemical markers and conduct laboratory tests. The samples were aliquoted and stored at –20 °C until analysis. Blood was divided as follows:

- A total of 2 mL in ethylenediaminetetraacetic acid (EDTA) tubes;
- A total of 6 mL in serum separator tubes (SST);
- A total of 2 mL in sodium fluoride tubes for fasting blood glucose measurements.

The SST samples were centrifuged at 3500 rpm for 10 min to analyze the following biochemical markers:

- Calcitonin Hormone: Measured using a Human Calcitonin (CT) kit, with a normal range of 4.5–40 pg/mL, via the DSX Automated ELISA Processing System.
- Parathyroid Hormone (PTH): Measured using a diagnostic spectrophotometer kit, with a normal range of 15–68.3 pg/mL, on the Abbott Architect C 8000 analyzer.
- Vitamin D: Measured using a diagnostic spectrophotometer kit, with a normal range of 20–30 ng/mL.
- Calcium Levels: Measured using a diagnostic spectrophotometer kit (lot number 44322UN21, Germany), with a normal range of 2.12–2.52 mmol/L.
- Albumin (ALB): Measured using a diagnostic spectrophotometer kit, with a normal range of 34–50 g/L. ALB-corrected calcium was calculated using the formula

Corrected calcium = measured calcium + {(40 − ALB) × 0.02}.

- Alkaline Phosphatase: Measured using a diagnostic spectrophotometer kit, with a normal range of 50–140 U/L.
- Estrogen Hormone: Measured using a diagnostic spectrophotometer kit, with a normal range of 12.5–166 pg/mL.

Additionally, 2 mL of whole blood in EDTA tubes was used for glycated hemoglobin (HbA1c) testing, with a normal range of 4.6–6%. Sodium fluoride samples were used for fasting blood glucose measurements, with a normal range of 90–100 mg/dL (3.89–6.05 mmol/L).

All laboratory analyses were performed at the central laboratory of Qassim University, adhering to internal and external quality control measures to ensure analytical accuracy and precision.

## 3. Statistical Analysis

Statistical analyses were conducted using IBM SPSS Statistics (Version 26). Data normality was tested using the Kolmogorov–Smirnov test. Continuous variables were expressed as mean ± standard deviation (SD), while categorical variables were reported as frequencies and percentages. Independent *t*-tests were used to compare mean differences between the diabetes and non-diabetes groups, while chi-square tests assessed categorical variable associations. A one-way ANOVA was conducted to compare BMD variations across subgroups, and Pearson correlation coefficients evaluated associations between BMD and biochemical markers. Multivariate regression analysis was employed to identify independent predictors of osteoporosis in women with and without diabetes. A *p*-value < 0.05 was considered statistically significant. To ensure scientific rigor, this study adhered to internationally recognized methodologies for assessing osteoporosis and metabolic bone health. Unlike previous studies, our research uniquely focuses on site-specific BMD variations (FN vs. LS), metabolic markers (PTH, ALP, estrogen), and the role of BMI in postmenopausal women with diabetes in the Qassim region. The inclusion of stringent participant selection criteria and advanced statistical modeling enhances the validity of our findings, providing novel insights into the pathophysiology of osteoporosis in T2DM populations.

## 4. Results

A total of 250 postmenopausal women were included in this study, with 150 participants diagnosed with T2DM and 100 controls without diabetes. Baseline characteristics, including age, menopausal duration, and BMI, were comparable between the groups, ensuring robust comparative analysis (Table 1).

### 4.1. Comparison of Clinical and Laboratory Characteristics Between the Postmenopausal Group with Diabetes and the Control Group (Table 2)

No statistically significant differences were observed between the postmenopausal group with diabetes and the control group in terms of menopause duration, age, vitamin D levels, calcitonin levels, parathyroid hormone (PTH) levels, albumin, body mass index (BMI), and lumbar spine (LS) T-score (*p* > 0.05). However, the diabetes group exhibited significantly higher levels of glycated hemoglobin (HbA1c) (8.81 ± 1.64), fasting blood glucose (FBG) (9.05 ± 3.56), and femoral neck (FN) T-score (−0.48 ± 1.07) compared to controls (*p* = 0.001). Conversely, alkaline phosphatase (ALP) (92.78 ± 20.27), estrogen levels (19.66 ± 17.2), and total calcium levels (2.32 ± 0.12) were significantly higher in the control group than in the diabetes group (*p* < 0.001), as detailed in Table 2.

**Table 2:** Comparison of clinical and laboratory characteristics between the postmenopausal group with diabetes and the control group.

Parameter Mean ± SD	Diabetes Patients N = 150 (Mean ± SD)	Control N = 100 (Mean ± SD)	*p*-Value
Duration of menopause (year)	8.78 ± 6.66	7.18 ± 6.01	0.054
Age (year)	58.47 ± 6.07	57.13 ± 6.77	0.103
HbA1c (%)	8.81 ± 1.64	5.33 ± 0.37	*** 0.000
Fasting blood glucose (mmol/L)	9.05 ± 3.56	5.13 ± 0.47	*** 0.000
Vit- D (ng/mL)	28.05 ± 22.43	23.56 ± 11.22	0.065
ALP(U/L)	85.45 ± 21.93	92.78 ± 20.27	** 0.008
Calcitonin(pg/mL)	10.8 ± 10	8.90 ± 5.161	0.063
PTH (pg/mL)	65.77 ± 26.22	68.65 ± 14.77	0.320
Estrogen (pg/mL)	12.47 ± 8.97	19.66 ± 17.2	*** 0.000
ALB (g/L)	40.81 ± 5.18	41.2 ± 2.62	0.488
BMI (kg/m^2^)	32.97 ± 5.18	33.6 ± 24.57	0.762
Total Calcium (mmol/L)	2.2 ± 0.18	2.32 ± 0.12	*** 0.000
T score Lumbar Spine (LS) (g/cm^2^)	−1.46 ± 1.27	−1.44 ± 1.04	0.918
T score Femoral Neck (FN) (g/cm^2^)	−0.48 ± 1.07	−0.91 ± 0.88	*** 0.001

Data are expressed as mean ± SD.; ** significance level at 0.01; *** significance level at 0.001; Alp: alkaline phosphatase; PTH: parathyroid hormone; Vit- D: vitamin D, ng/mL (using the T-Test).

### 4.2. Bone Mineral Density (BMD) Differences Between the Diabetes Group and the Control Group (Table 3)

The analysis of BMD (DXA scan) results showed no statistically significant differences in the prevalence of osteoporosis, osteopenia, and normal BMD between the diabetes group and the control group, as determined by a chi-square test (*p* > 0.05) (Table 3).

**Table 3:** Differences in BMD (DXA SCAN) between the postmenopausal group with diabetes and the control group (percentage of osteoporosis, osteopenia, and normal) (chi-square test X^2^ & *p*-value).

DXA SCAN	Groups			*p*
	Diabetes Patients	Controls	Chi-Square	
DXA Scan Lumbar Spine				
NORMAL	37.3%	40.0%		NS
OSTEOPENIA	40.7%	42.0%	0.608	
OSTEOPOROSIS	22.0%	18.0%		
DXA Scan Femoral Neck				
NORMAL	68.7%	65.0%		NS
OSTEOPENIA	29.3%	31.0%	1.033	
OSTEOPOROSIS	2.0%	4.0%		

Data are expressed as percentages; NS indicates no significance as assessed by the chi-square test (X^2^).

### 4.3. Clinical and Laboratory Characteristics Based on DXA Scan of FN and LS (Table 4)

Among diabetes patients with osteoporosis (OP) at the femoral neck (FN), there was a significant increase in menopause duration (16.33 ± 20.65 years), age (67.67 ± 8.14 years), ALP (115.67 ± 20.26 U/L), PTH (103.1 ± 79.1 pg/mL), and total calcium (2.27 ± 0.32 mmol/L), along with a significant reduction in estrogen (8.53 ± 6.11 pg/mL), albumin (35.87 ± 1.86 g/L), BMI (30.28 ± 4.97 kg/m^2^), and LS T-score (−3.57 ± 0.49). In contrast, the control group with OP showed significantly higher PTH levels (76.14 ± 12.4 pg/mL) and significantly lower vitamin D (17.93 ± 10.28 ng/mL), estrogen (15.5 ± 10.75 pg/mL), and LS T-scores (−2.22 ± 0.99).

Regarding osteoporosis at the lumbar spine (LS) in diabetes patients, menopause duration (12.06 ± 8.27 years) and ALP levels (91.82 ± 22.86 U/L) were significantly elevated, whereas estrogen (8.83 ± 5.29 pg/mL), BMI (31.29 ± 5.78 kg/m^2^), and FN T-scores (−1.49 ± 0.71) were significantly reduced. No other significant differences were noted in this group. In the control group with OP, PTH levels were significantly increased (81.36 ± 7.71 pg/mL), while vitamin D (17.68 ± 7.35 ng/mL) and estrogen (19.7 ± 11 pg/mL) were significantly decreased.

**Table 4:** The clinical and laboratory characteristics of the postmenopausal group with diabetes and the control group based on DXA of the femoral neck and lumbar spine.

Mean ± SD	DXA Scan Femoral Neck (Diabetes Patients)			*p*	DXA Scan Femoral Neck (Controls)			*p*
	NORMAL	OST.ENIA	OST.SIS		NORMAL	OST.ENIA	OST.SIS	
**Menopause Duration (Years)**	7.2 ± 5.23	11.95 ± 6.97	16.33 ± 20.65	*** <0.001	7.35 ± 6.67	6.77 ± 4.13	7.5 ± 8.5	**NS**
**Age (years)**	57.03 ± 5.42	61.23 ± 6.03	67.67 ± 8.14	*** <0.001	56.29 ± 6.93	58.48 ± 5.1	60.25 ± 13.43	**NS**
**HbA1c (%)**	8.93 ± 1.66	8.62 ± 1.6	7.57 ± 0.63	NS	5.3 ± 0.39	5.39 ± 0.32	5.25 ± 0.53	**NS**
**FBG (mmol/L)**	9.1 ± 3.58	9.02 ± 3.64	8.01 ± 1.17	NS	5.18 ± 0.49	5.01 ± 0.43	5.13 ± 0.52	**NS**
**Vit- D(ng/mL)**	26.9 ± 16.56	31.52 ± 32.62	16.43 ± 8.6	NS	24.28 ± 11.61	22.76 ± 10.54	17.93 ± 10.28	** <0.01
**ALP(U/L)**	83.77 ± 21.91	87.34 ± 20.83	115.67 ± 20.26	* <0.05	94.05 ± 21.43	89.0 ± 17.94	101.5 ± 16.22	**NS**
**Calcitonin** **(pg/mL)**	10.91 ± 10.46	10.95 ± 9.18	4.77 ± 0.71	NS	8.680 ± 5.32	9.971 ± 5.09	10.325 ±2.779	**NS**
**PTH (pg/mL)**	65.93 ± 26.02	62.84 ± 19.47	103.1 ± 79.1	* <0.05	64.97 ± 14.39	70.3 ± 18.469	76.14 ± 12.4	** <0.01
**Estrogen (pg/mL)**	14.05 ± 10.0	9.05 ± 4.62	8.53 ± 6.11	** <0.01	20.22 ± 18.06	19.02 ± 16.29	15.5 ± 10.75	** <0.01
**ALB (g/L)**	42.52 ± 3.65	40.23 ± 5.58	35.87 ± 1.86	* <0.05	41.31 ± 2.7	40.8 ± 2.28	42.59 ± 3.84	**NS**
**BMI (kg/m^2^)**	33.74 ± 5.08	31.34 ± 5.07	30.28 ± 4.97	* <0.05	35.21 ± 30.27	30.96 ± 4.4	27.93 ± 3.29	**NS**
**Total Calcium (mmol/L)**	2.22 ± 0.18	2.14 ± 0.16	2.27 ± 0.32	* <0.05	2.31 ± 0.12	2.36 ± 0.12	2.32 ± 0.03	**NS**
**T score LS (g/cm^2^)**	−0.99 ± 1.15	−2.42 ± 0.8	−3.57 ± 0.49	*** <0.001	−1.05 ± 0.76	−1.78 ± 1.94	−2.22 ± 0.99	*** <0.001
	**DXA Scan Lumbar Spine (diabetes patients)**			***p***	**DXA Scan Lumbar Spine (controls)**			***p***
**Parameter** **mean ± SD**	NORMAL	OST.ENIA	OST.SIS		NORMAL	OST.ENIA	OST.SIS	
**Menopause duration (year)**	6.82 ± 5.69	8.8 ± 5.86	12.06 ± 8.27	*** <0.001	6.58 ± 6.53	7.95 ± 6.2	6.72 ± 4.2	**NS**
**Age (years)**	57.32 ± 5.23	58.67 ± 6.79	60.06 ± 5.76	NS	55.88 ± 6.95	57.69 ± 6.83	58.61 ± 6.04	**NS**
**HbA1c (%)**	9.05 ± 1.72	8.73 ± 1.7	8.56 ± 1.35	NS	5.28 ± 0.37	5.42 ± 0.39	5.22 ± 0.28	**NS**
**FBG (mmol/L)**	9.15 ± 3.43	8.63 ± 3.63	9.67 ± 3.63	NS	5.16 ± 0.51	5.2 ± 0.48	4.89 ± 0.29	**NS**
**Vit-D (ng/mL)**	24.92 ± 15.13	30.39 ± 26.35	29.02 ± 24.87	NS	24.55 ± 13.04	21.13 ± 9.06	17.68 ± 7.35	* <0.05
**ALP(U/L)**	80.07 ± 21.41	86.95 ± 21.06	91.82 ± 22.86	* <0.05	97.2 ± 18.89	89.12 ± 22.79	91.5 ± 15.52	**NS**
**Calcitonin (pg/mL)**	10.94 ± 9.79	9.7 ± 8.95	12.59 ± 12.05	NS	7.732 ± 4.137	9.819 ± 5.663	9.856 ± 5.362	**NS**
**PTH (pg/mL)**	65.19 ± 24.72	63.76 ± 21.28	70.45 ± 35.66	NS	57.92 ± 10.66	73.41 ± 13.69	81.36 ± 7.71	*** <0.001
**Estrogen (pg/mL)**	15.24 ± 10.94	11.82 ± 7.72	8.83 ± 5.29	** <0.01	25.71 ± 23.83	19.7 ± 11	13.87 ± 7.35	** <0.01
**ALB (g/L)**	40.5 ± 5.53	41.36 ± 4.61	40.33 ± 5.61	NS	41.46 ± 3.05	41.2 ± 2.37	40.63 ± 2.17	**NS**
**BMI(kg/m^2^)**	34.54 ± 4.24	32.44 ± 5.3	31.29 ± 5.78	** <0.01	30.96 ± 4.67	37.37 ± 37.49	30.64 ± 3.9	**NS**
**Total Calcium (mmol/L)**	2.22 ± 0.2	2.19 ± 0.17	2.17 ± 0.16	NS	2.31 ± 0.09	2.32 ± 0.14	2.36 ± 0.09	**NS**
**T score FN** **(g/cm^2^ )**	**0.3 ± 0.94**	**−0.65 ± 0.76**	**−1.49 ± 0.71**	*** <0.001	**6.58 ± 6.53**	**7.95 ± 6.2**	**6.72 ± 4.2**	**NS**

Data are expressed as mean ± SD. NS indicates not significant; * significance level at 0.05; ** significance level at 0.01; *** significance level at 0.001. Alp: alkaline phosphatase (using one-way ANOVA test).

### 4.4. Factors Associated with BMD in DXA Scan of FN and LS (Table 5)

Multivariate analysis identified significant predictors of OP in both groups. In postmenopausal diabetes patients, menopause duration (B = 0.117, *p* = 0.003) and estrogen levels (B = −0.113, *p* = 0.006) were significantly associated with OP, while no significant associations were found for osteopenia. In contrast, in the control group, FBG (B = −2.856, *p* = 0.012) and vitamin D (B = −0.140, *p* = 0.009) were negatively correlated with OP, whereas calcitonin (B = 1.341, *p* = 0.002) and PTH (B = 0.185, *p* < 0.001) showed positive associations. Additionally, osteopenia in the control group was significantly influenced by estrogen (B = −0.139, *p* = 0.003, negative correlation), calcitonin (B = 1.021, *p* = 0.006, positive correlation), and PTH (B = 0.108, *p* < 0.001, positive correlation) (Table 5).

**Table 5:** Multivariate logistic regression analysis to determine the influence of the independent variables associated with DXA scan of the lumbar spine and FN.

DXA Scan Lumbar Spine. a			B	Sig.	Exp(B)	*95% CI Exp(B)*	
						Lower Bound	Upper Bound
***Diabetes patients***	OSTEOPENIA	Intercept	0.152	0.722			
		Duration of menopause	0.060	0.081	1.062	0.993	1.135
		Estrogen	−0.039	0.064	0.961	0.922	1.002
	OSTEOPOROSIS	Intercept	−0.335	0.564			
		Duration of menopause	0.117	0.003	1.124	1.042	1.212
		Estrogen	−0.113	0.006	0.893	0.825	0.968
***Controls***	OSTEOPENIA	Intercept	−4.127	0.286			
		FBG	−0.388	0.555	0.679	0.187	2.461
		Vit-D	−0.042	0.144	0.959	0.907	1.014
		Calcitonin	1.021	0.006	2.775	1.347	5.717
		PTH	0.108	0.000	1.114	1.057	1.174
		Estrogen	−0.139	0.003	0.870	0.794	0.953
	OSTEOPOROSIS	Intercept	1.334	0.835			
		FBG	−2.856	0.012	0.058	0.006	0.529
		Vit-D	−0.140	0.009	0.870	0.783	0.966
		Calcitonin	1.341	0.002	3.823	1.620	9.020
		PTH	0.185	0.000	1.204	1.101	1.316
		Estrogen	−0.073	0.161	0.929	0.839	1.029
**DXA Scan Femoral Neck. a**			B	Sig.	Exp(B)	95% CI Exp(B)	
						Lower Bound	Upper Bound
**Diabetes patients**	OSTEOPENIA	Duration of menopause	0.117	0.000	1.125	1.058	1.196
		Age	0.126	0.000	1.134	1.062	1.212
		Estrogen	−0.098	0.003	0.907	0.850	0.967
		ALB	0.109	0.016	1.115	1.021	1.218
		BMI	−0.100	0.011	0.905	0.837	0.978
		Total calcium	−2.860	0.016	0.057	0.006	0.583
	OSTEOPOROSIS	Duration of menopause	0.174	0.009	1.191	1.044	1.358
		Age	0.296	0.007	1.345	1.086	1.665
		ALP	0.065	0.034	1.067	1.005	1.134
		PTH	0.029	0.039	1.029	1.001	1.057
**Controls**	OSTEOPENIA	PTH	0.061	0.001	1.063	1.025	1.103
	OSTEOPOROSIS	Calcitonin	0.923	0.021	2.517	1.151	5.505

DXA Scan (NORMAL, OSTEOPENIA, and OSTEOPOROSIS); the reference category is NORMAL (as studied using multivariate logistic regression analysis).

Regarding FN BMD, six variables were significantly associated with OP in the diabetes group: menopause duration (B = 0.117, *p* < 0.001), age (B = 0.126, *p* < 0.001), and albumin levels (B = 0.109, *p* = 0.016) were positively correlated, whereas estrogen levels (B = −0.098, *p* = 0.003), BMI (B = −0.100, *p* = 0.011), and total calcium levels (B = −2.86, *p* = 0.016) showed negative associations. Additionally, OP in the diabetes group was significantly influenced by menopause duration (B = 0.174, *p* = 0.009), age (B = 0.296, *p* = 0.007), albumin (B = 0.065, *p* = 0.034), and PTH (B = 0.029, *p* = 0.039). In the control group, OP was positively associated with calcitonin (B = 0.923, *p* = 0.021), while osteopenia correlated positively with PTH (B = 0.061, *p* = 0.001) (Table 5).

### 4.5. Correlation Between BMD (LS and FN T-Score) and HbA1c in Postmenopausal Women with and Without Diabetes (Figure 1 and Figure 2)

**Figure 1:**
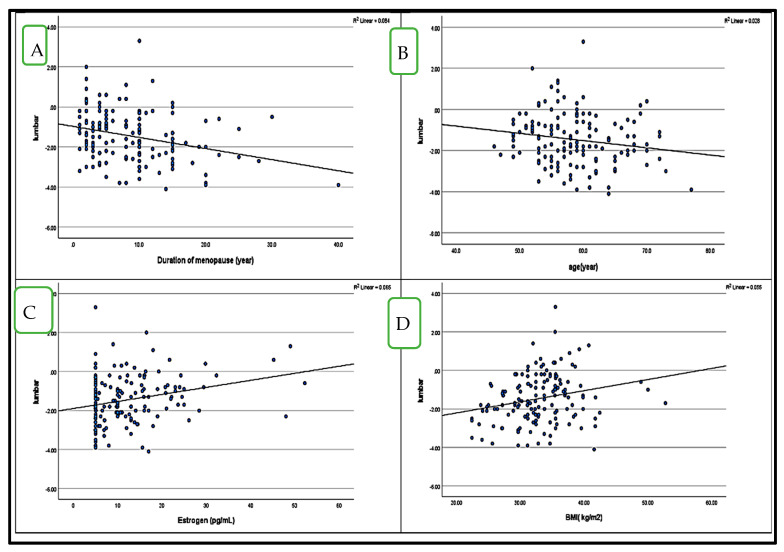
Significant correlations between LS T score, a dependent variable, and other variables in postmenopausal diabetes patients: (**A**); menopause duration (r = − 0.291), (**B**); age (r = − 0.168), (**C**); estrogen (r = 0.256), (**D**); BMI (r = 0.236).

Pearson’s correlation analysis demonstrated a strong direct correlation between LS T-scores and FN T-scores in the diabetes group (r = 0.726, *p* = 0.01) and a moderate direct correlation in the control group (r = 0.505, *p* = 0.01). In the diabetes group, LS T-scores were significantly negatively correlated with menopause duration (r = −0.291, *p* = 0.01) and age (r = −0.168, *p* = 0.05), while positive correlations were observed with estrogen levels (r = 0.256, *p* = 0.01) and BMI (r = 0.236, *p* = 0.01) (Figure 1).

**Figure 2:**
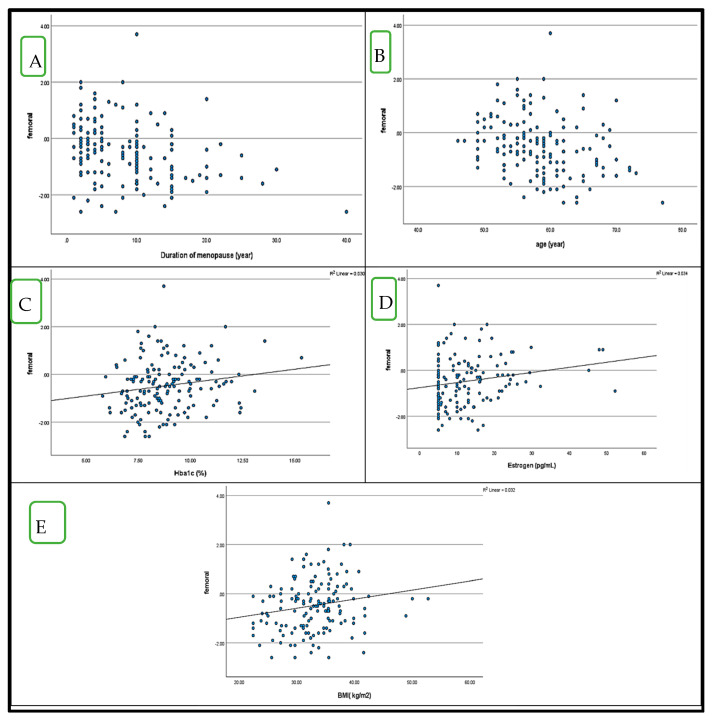
Correlations between FN T score, a dependent variable, and other variables in postmenopausal diabetes patients.; (**A**), duration of menopause (r =0.322), (**B**); age (r = −0.288), (**C**); HbA1c % (r = 0.173), (**D**); estrogen (r = 0.185). (**E**); BMI (r = 0.179).

Similarly, FN T-scores showed significant negative correlations with menopause duration (r = −0.322, *p* = 0.01) and age (r = −0.288, *p* = 0.01), while positive correlations were noted with estrogen levels (r = 0.185, *p* = 0.05), HbA1c % (r = 0.173, *p* = 0.05), and BMI (r = 0.179, *p* = 0.05). Additionally, HbA1c % had a strong positive correlation with FBG levels (r = 0.668, *p* = 0.01) and a weak positive correlation with ALP levels (r = 0.247, *p* = 0.01) (Figure 2). In the control group, LS T-scores were moderately negatively correlated with PTH (r = −0.485, *p* = 0.01), while FN T-scores had weak inverse correlations with PTH and age (r = −0.219, r = −0.216, *p* = 0.05, respectively). Furthermore, HbA1c showed a moderate positive correlation with FBG (r = 0.367, *p* = 0.01).

These findings suggest that T2DM is associated with site-specific variations in BMD, rather than a universal reduction in bone density. The observed protective effect of BMI on FN BMD aligns with previous reports indicating that mechanical loading contributes to improved bone strength. However, the increased PTH and ALP levels in osteoporotic patients highlight a potential compensatory mechanism in bone turnover, warranting further investigation (Figure 2).

Overall, this study underscores the complexity of the relationship between T2DM and osteoporosis, emphasizing the need for a multidimensional approach when assessing bone health in postmenopausal women with diabetes. Future longitudinal studies are necessary to explore the causative pathways underlying these associations and to develop targeted intervention strategies.

## 5. Discussion

The relationship between T2DM and osteoporosis remains a topic of ongoing debate, with conflicting findings regarding the impact of diabetes on BMD and fracture risk. While T2DM has been associated with alterations in BMD and increased fracture risk, the exact mechanisms underlying these effects remain unclear. Earlier research has documented varying prevalence rates of OP among postmenopausal Saudi women with T2DM, ranging from 29.4% to 34% [13]. While some studies suggest that T2DM patients exhibit increased or preserved BMD despite an elevated fracture risk [1,4], others indicate a significant reduction in BMD, particularly among postmenopausal women [14,15]. This study aimed to address this discrepancy by evaluating BMD variations in postmenopausal Saudi women with and without T2DM, shedding light on potential mechanisms contributing to bone fragility in this population. A total of 250 postmenopausal women were recruited from the Qassim region, Saudi Arabia, including 150 individuals with T2DM and 100 controls without diabetes. BMD was assessed at the LS and FN using DXA. Based on BMD results, participants were categorized into normal, osteopenia, and OP groups. Statistical analysis revealed no significant differences in LS and FN BMD between the groups with and without diabetes. However, the FN T-score was significantly higher in T2DM patients compared to controls, suggesting a potential protective effect of diabetes on BMD at this site. Our results align with previous studies that have reported increased or preserved BMD in T2DM patients despite their elevated risk of fragility fractures [1,2,13].

In contrast, studies by Al-Maatouq et al. [14], Zeid et al. [15], and Roomi et al. [16] have documented reduced BMD in postmenopausal women with T2DM, underscoring the ongoing controversy regarding the impact of diabetes on bone health. These conflicting results highlight the need for further research to elucidate the mechanisms linking T2DM, bone quality, and fracture susceptibility in postmenopausal women. The present study contributes to this discourse by demonstrating that while T2DM is associated with a significantly higher FN T-score than controls without diabetes, LS BMD remains largely unaffected. These findings reinforce the notion that the effects of diabetes on bone health may be site-specific, rather than generalized, which has important implications for the assessment and management of osteoporosis in postmenopausal women with diabetes [4,5].

Bone metabolism is closely linked to glucose metabolism and HbA1c [17]. Insulin plays a vital role in maintaining bone density and preventing bone loss [18]. Reduced insulin secretion, commonly observed in T2DM, may contribute to OP development [18]. However, conflicting data exist regarding the association between insulin resistance and increased OP risk [17,18,19]. The disruption of bone metabolism due to altered cytokine secretion in T2DM may contribute to bone mass loss [19]. Wang et al. [20] suggested that bone resorption is lower in T2DM patients than in individuals without diabetes, yet the direct effect of insulin on bone cells remains unclear. While increased circulating insulin has been associated with greater FN bone mass, the exact mechanisms underlying this relationship require further investigation [21].

The current study findings suggest that T2DM does not directly cause OP. The influence of glycemic control on bone turnover in T2DM remains uncertain; however, some studies have reported a significant reduction in BMD in patients with poorly controlled diabetes [22]. Given the ongoing debate regarding the reliability of BMD in assessing fracture risk in T2DM, it is imperative to explore additional markers of bone health. Previous research has highlighted a greater reduction in FN BMD than in LS BMD in T2DM patients [23]. In contrast, Majima et al. [23] found no significant differences in FN and LS BMD between individuals with and without diabetes. Furthermore, this research uniquely highlights the lack of association between FBG, HbA1c, and BMD, challenging conventional assumptions about the direct role of hyperglycemia in bone deterioration. While some studies have linked poor glycemic control with lower BMD and increased fracture risk [2,9], our findings suggest that additional mechanisms—such as chronic inflammation, oxidative stress, and advanced glycation end-products (AGEs)—may play a more substantial role in osteoporosis in individuals with diabetes [8,11]. This underscores the need for a paradigm shift in how osteoporosis risk is assessed in diabetes patient populations, advocating for the inclusion of bone turnover markers and advanced imaging techniques to better predict fracture susceptibility.

This paradoxical finding has been attributed to several factors, including the potential anabolic effects of hyperinsulinemia on bone mass and the mechanical loading associated with higher BMI, which is frequently observed in populations with diabetes [3]. Additionally, this study reinforces the protective role of BMI in FN BMD, supporting previous research indicating that mechanical loading from higher body weight may contribute to bone strength. However, it also raises concerns about whether this protective effect is sufficient to offset the detrimental metabolic changes associated with diabetes-related bone loss [9]. However, despite higher BMD, diabetes patients still face an elevated fracture risk due to compromised bone microarchitecture and an increased susceptibility to falls, underscoring the importance of assessing bone quality beyond BMD alone [4].

A strong inverse relationship between age and BMD has been widely reported [12]. Our results indicate that age is a significant predictor of OP and osteopenia. The age-related decline in BMD is likely due to decreased calcium absorption and hormonal alterations associated with aging [24]. The role of estrogen deficiency in postmenopausal osteoporosis is well established, and our findings further emphasize its critical role in maintaining bone homeostasis, particularly in individuals with diabetes [10].

Estrogen plays a crucial role in maintaining trabecular bone mass via estrogen receptor signaling. Following menopause, estrogen depletion disrupts vitamin D receptor (VDR) function, increasing the body’s requirement for vitamin D and calcium [25]. Vitamin D levels demonstrated an inverse correlation with age and HbA1c in postmenopausal women [26,27]. Aging impairs intestinal vitamin D absorption and reduces cutaneous vitamin D synthesis [28]. Furthermore, declining estrogen levels in postmenopausal women impair the activity of 1-alpha hydroxylase, the enzyme responsible for vitamin D activation [26,27,28].

Analysis of independent variables associated with DXA scan FN and LS revealed that menopause duration was positively correlated with OP, making it a significant predictor of progression from osteopenia to OP. These findings corroborate those of Sharifi et al. [29] and Hyassat et al. [30].

By incorporating biochemical markers such as PTH, ALP, and estrogen levels into our analysis, our study also provides a more comprehensive metabolic profile of bone health in postmenopausal women with diabetes. The significant inverse correlation between PTH and femoral neck BMD highlights a potential compensatory mechanism in bone metabolism in diabetes, an area that remains underexplored in the current literature [10]. In contrast, LS BMD did not show a significant association with these metabolic markers, reinforcing the hypothesis that diabetes-related alterations in bone metabolism may affect different skeletal sites in distinct ways [8]. Majima et al. [23] suggested that cortical bone loss in T2DM patients may be attributed to secondary hyperparathyroidism resulting from calcium imbalance. While previous studies have documented that T2DM is associated with either preserved or increased BMD, particularly at weight-bearing sites such as the FN, our study challenges this notion by demonstrating that despite a higher FN T-score in diabetes patients, significant metabolic imbalances—including elevated PTH and ALP levels—may counteract any perceived protective effect of diabetes on bone health [5]. This aligns with global research indicating that T2DM patients remain at an increased risk of fragility fractures due to impaired bone quality, even when BMD appears to be normal or increased [4]. This study adds to the growing body of evidence suggesting that traditional BMD assessments may not be sufficient in evaluating fracture risk in individuals with diabetes 1. Our results support this hypothesis, as individuals with diabetes with FN OP and individuals without diabetes with LS OP exhibited significant PTH levels, consistent with findings from previous studies [27,29,30].

Multivariate regression analysis identified menopause duration, PTH, ALP, and estrogen levels as independent predictors of osteoporosis in women with diabetes, while BMI emerged as a protective factor. These findings highlight the multifaceted nature of osteoporosis in T2DM, suggesting that bone health in postmenopausal women with diabetes is influenced by a combination of hormonal, metabolic, and biomechanical factors. Notably, the absence of a significant predictive role for HbA1c further underscores the complexity of the diabetes–osteoporosis relationship, reinforcing the need for a more comprehensive approach to bone health assessment in diabetes patients [6].

The observed site-specific differences in BMD warrant further exploration to elucidate the underlying pathophysiological mechanisms driving these variations. One possible explanation is that the trabecular-rich structure of the lumbar spine may be more susceptible to diabetes-related changes in bone quality, whereas the predominantly cortical composition of the femoral neck may be less affected [5]. This study provides a novel contribution to the ongoing discourse on the relationship between T2DM and OP by offering a site-specific analysis of BMD variations among postmenopausal women in a Middle Eastern population. Unlike prior studies that have primarily focused on Caucasian or Asian cohorts, our research uniquely examines the interplay between metabolic factors and BMD in Saudi Arabian postmenopausal women, a demographic often under-represented in global OP research [11]. Given the high prevalence of both T2DM and OP in the region, our findings contribute to a deeper understanding of how ethnicity, lifestyle, and genetic predisposition influence bone health in individuals with diabetes.

This study contributes novel insights by demonstrating that the apparent protective effect of T2DM on femoral neck BMD is potentially outweighed by underlying metabolic imbalances, reinforcing the need for a more nuanced approach to osteoporosis assessment in individuals with diabetes. This study underscores the importance of integrating metabolic markers and alternative bone quality assessments to predict fracture risk efficiently, ultimately challenging current clinical guidelines, which rely primarily on BMD measurements. By comparing our findings with global studies and emphasizing regional specificities, this study enhances the understanding of bone health in diabetes patients and calls for a more tailored approach to OP prevention in high-risk populations. Moreover, our study fills a critical gap in Middle Eastern osteoporosis research by providing region-specific data that can be used to guide targeted intervention strategies. Given the distinct dietary habits, vitamin D status, and lifestyle factors in Saudi Arabia, current findings highlight the necessity of localized public health policies aimed at OP screening and management among postmenopausal women with diabetes [6]. Future studies should further explore the role of genetic predisposition and dietary influences on bone health in T2DM patients from diverse ethnic backgrounds.

### Limitations and Recommendation

Despite the strengths of this study, its cross-sectional design limits the ability to establish a causal relationship between T2DM and OP. Longitudinal studies are needed to investigate the long-term impact of diabetes on bone health and fracture risk. Although BMD was assessed using dual-energy X-ray absorptiometry, this method does not directly evaluate bone quality, a crucial factor influencing fracture risk in patients with diabetes. Future studies should incorporate advanced imaging techniques such as high-resolution peripheral quantitative computed tomography (HR-pQCT) and Trabecular Bone Score (TBS) to provide a more comprehensive assessment of bone microarchitecture. Additionally, this study did not assess bone remodeling biomarkers such as C-terminal telopeptide of type I collagen (CTX) and procollagen type 1 N-terminal propeptide (P1NP), which are important indicators of bone turnover and should be considered in future research.

## 6. Conclusions

This study highlights the complex interplay between T2DM and osteoporosis, emphasizing the site-specific nature of BMD alterations and the role of hormonal and metabolic factors in bone health. Our findings suggest that while BMI may exert a protective effect on FN BMD, diabetes-related alterations in bone turnover and estrogen deficiency contribute to osteoporosis risk. The lack of a significant relationship between HbA1c and BMD challenges traditional assumptions about glycemic control and bone health, underscoring the need for further research into alternative mechanisms affecting skeletal integrity in patients with diabetes. Given the limitations of cross-sectional analyses, longitudinal studies are essential to better understand the long-term impact of diabetes on bone health and fracture risk. These insights have important clinical implications for the early identification and management of osteoporosis in postmenopausal women with diabetes, reinforcing the need for tailored screening and intervention strategies beyond conventional BMD assessment.

## Figures and Tables

**Table 1:** The general characteristics of the postmenopausal group with diabetes and the control group.

		Diabetes Patients N = 150	Control N = 100	*p*- Value
**1**	**Marital status:**			
	Widower	46 (30.67%)	21 (21%)	
	Married	100 (66.67%)	76 (76%)	**NS**
	Divorced	4 (2.67%)	3 (3%)	
**2**	**Educational level:**			
	No education	88 (58.67%)	39 (39%)	
	Primary	41 (27.33%)	27 (27%)	
	Middle education	12 (8%)	13 (13%)	**NS**
	Secondary	4 (2.67%)	10 (10%)	
	University and above	5 (3.33%)	11 (11%)	
**4**	**Do you have any medical history?**			
	No	132 (88%)	93 (93%)	**NS**
	Yes	18 (12%)	7 (7%)	
**5**	**Do you have any smoking history?**			
	No	150 (100%)	99 (99%)	**NS**
	Yes	0 (0%)	1 (1%)	
**6**	**Do you practice a physical activity?**			
	No	43 (28.67%)	24 (24%)	**NS**
	Walking sport	107 (71.33%)	76 (76%)	
**7**	**Do you consume calcium daily?**			
	No	28 (18.67%)	14 (14%)	**NS**
	Yes	122 (81.33%)	86 (86%)	
**8**	**Are you exposed to sunlight?**			
	No	52 (34.67%)	36 (36%)	**NS**
	Yes	98 (65.33%)	64 (64%)	
**9**	**Do you drink caffeine?**			
	No	4 (2.67%)	7 (7%)	**NS**
	Yes	146 (97.33%)	93 (93%)	
**10**	**Does family history include osteoporosis or fragility fracture?**			
	No	137 (91.33%)	80 (80%)	**NS**
	Yes	13 (8.67%)	20 (20%)	
**11**	**Do you have osteoporosis?**			
	No	126 (84%)	94 (94%)	**NS**
	Yes	24 (16%)	6 (6%)	
**12**	**Breastfeeding**			
	No	6 (4%)	5 (5%)	**NS**
	Yes	144 (96%)	95 (95%)	
**13**	**Diabetes status**			
	Gestational diabetes	37 (24.67%)	0 (0%)	
	Diabetes	113 (75.33%)	0 (0%)	**NS**
	Nothing	0 (0%)	100 (100%)	
**14**	**Duration of Diabetes status (years)**	15.06 ± 7.84	0	
**15**	**Hypertension status**			
	Hypertension	70 (46.67%)	18 (18%)	
	Hypotension	0 (0%)	1 (1%)	**NS**
	Nothing	80 (53.33%)	81 (81%)	
**16**	**Use of statin or vitamin D3 supplementation:**			
	Every week	32 (21.33%)	15 (15%)	
	Per month	3 (2%)	2 (2%)	**NS**
	Every day	10 (6.67%)	3 (3%)	
	**Nothing**	**105 (70%)**	**80 (80%)**	

Data are presented as numbers (percentage) for categorical data and (mean ± standard deviation) for parametrically distributed data; NS indicates not significant (using the chi-square test X^2^).

## Data Availability

Due to participant consent agreements, the data collected and analyzed in this study are not publicly available. However, the corresponding author may access them upon reasonable request.
